# Modification of Heat-Related Mortality in an Elderly Urban Population by Vegetation (Urban Green) and Proximity to Water (Urban Blue): Evidence from Lisbon, Portugal

**DOI:** 10.1289/ehp.1409529

**Published:** 2015-11-13

**Authors:** Katrin Burkart, Fred Meier, Alexandra Schneider, Susanne Breitner, Paulo Canário, Maria João Alcoforado, Dieter Scherer, Wilfried Endlicher

**Affiliations:** 1 Climatological Section, Geography Department, Humboldt-Universität zu Berlin, Berlin, Germany; 2 Department of Environmental Science, Mailman School of Public Health, Columbia University, New York, New York, USA; 3 Technische Universität Berlin, Department of Ecology, Berlin, Germany; 4 Institute of Epidemiology II, Helmholtz Zentrum München, German Research Center for Environmental Health (GmbH), Neuherberg/Oberschleißheim, Germany; 5 Universidade de Lisboa, IGOT, Centro de Estudos Geográficos, Lisbon, Portugal

## Abstract

**Background:**

Urban populations are highly vulnerable to the adverse effects of heat, with heat-related mortality showing intra-urban variations that are likely due to differences in urban characteristics and socioeconomic status.

**Objectives:**

We investigated the influence of urban green and urban blue, that is, urban vegetation and water bodies, on heat-related excess mortality in the elderly > 65 years old in Lisbon, Portugal, between 1998 and 2008.

**Methods:**

We used remotely sensed data and geographic information to determine the amount of urban vegetation and the distance to bodies of water (the Atlantic Ocean and the Tagus Estuary). Poisson generalized additive models were fitted, allowing for the interaction between equivalent temperature [universal thermal climate index (UTCI)] and quartiles of urban greenness [classified using the Normalized Difference Vegetation Index (NDVI)] and proximity to water (≤ 4 km vs. > 4 km), while adjusting for potential confounders.

**Results:**

The association between mortality and a 1°C increase in UTCI above the 99th percentile (24.8°C) was stronger for areas in the lowest NDVI quartile (14.7% higher; 95% CI: 1.9, 17.5%) than for areas in the highest quartile (3.0%; 95% CI: 2.0, 4.0%). In areas > 4 km from water, a 1°C increase in UTCI above the 99th percentile was associated with a 7.1% increase in mortality (95% CI: 6.2, 8.1%), whereas in areas ≤ 4 km from water, the estimated increase in mortality was only 2.1% (95% CI: 1.2, 3.0%).

**Conclusions:**

Urban green and blue appeared to have a mitigating effect on heat-related mortality in the elderly population in Lisbon. Increasing the amount of vegetation may be a good strategy to counteract the adverse effects of heat in urban areas. Our findings also suggest potential benefits of urban blue that may be present several kilometers from a body of water.

**Citation:**

Burkart K, Meier F, Schneider A, Breitner S, Canário P, Alcoforado MJ, Scherer D, Endlicher W. 2016. Modification of heat-related mortality in an elderly urban population by vegetation (urban green) and proximity to water (urban blue): evidence from Lisbon, Portugal. Environ Health Perspect 124:927–934; http://dx.doi.org/10.1289/ehp.1409529

## Introduction

Weather and climate exert far-reaching effects on human health and well-being. Given the projected consequences of climate change, the hazardous nature of heat effects and heat-related excess mortality have received increasing attention in science and politics. Taking into account the urban heat island effect, which is likely to aggravate health threats, urban areas are particularly vulnerable to heat stress (Burkart et al. 2011, 2014; Clarke 1972; Gabriel and Endlicher 2011; Schuman 1972; Smargiassi et al. 2009; Smoyer et al. 2000; Tan et al. 2010). The shape and magnitude of the urban heat island (UHI) differs throughout the urban landscape. While bigger and more densely built and populated urban areas generally show higher excess temperatures than do their rural surroundings, there are also intra-city variations in the UHI (Lopes et al. 2013; Oke 1973; Saaroni et al. 2000; Streutker 2003; Unger 2004). The formation of such micro- and meso-climates is a consequence of different mechanisms competing in the urban setting. Studies have highlighted the importance of large-scale geographic factors such as altitude, topography, latitude, and location relative to land and sea, as well as the influence of the urban structure and characteristics such as the percentage of built-up areas, the sky-view factor, and building height (Alcoforado and Andrade 2006; Alcoforado et al. 2014; Andrade and Alcoforado 2008; Kuttler 1998, 2004; Landsberg 1981; Oke 1973, 1987; Unger 2004).

Furthermore, the role of urban vegetation in shaping thermal environments has been highlighted in the literature. Vegetation mainly influences the micro- and meso-climate through shading and evapotranspiration (Andrade and Vieira 2007; Bernatzky 1982; Endlicher 2012; Georgi and Dimitriou 2010; Huang et al. 1987; Oliveira et al. 2011; Santamouris 2001; Souch and Souch 1993). The shading effect of vegetation reduces the incident shortwave radiation and leads to lower ground and wall surface temperatures. The effectiveness of shading depends on plant geometry (e.g., crown shape) and density. In addition, plants have a cooling effect through evapotranspiration. Rather than increasing the sensible heat flux (i.e., the air temperature), solar energy is transformed into latent heat by the transition of water from liquid to gas. In addition to the mitigating nature of urban vegetation, trees and shrubs might also act as windbreakers, increasing surface roughness and thereby reducing wind speed. During the summer, this effect might be unfavorable as air movement might be restricted thus leading to decreased cooling through wind.

The urban climate can also be influenced by bodies of water and coastal proximity. Generally, large water bodies have a mitigating influence on the climate, as displayed by more homogenous diurnal and seasonal temperature distributions (Schönwiese 2008; Weischet and Endlicher 2012). Moreover, during stable high-pressure conditions without large-scale air movement, coastal areas are usually dominated by a convective circulation system generated by temperature and pressure differences between the land and the sea, that is, the land-sea breeze which potentially plays an important role in heat mitigation (Alcoforado et al. 2009; Häckel 2008; Lopes et al. 2013; Papanastasiou et al. 2010). The Atlantic Ocean and Tagus Estuary breezes play an important role in the urban climate of Lisbon, notably in the ventilation of the waterside districts, where air temperatures are up to 3–4°C lower than in the city center (Alcoforado et al. 2009; Vasconcelos and Lopes 2006).

Cities in Southern Europe have been shown to be highly vulnerable to heat with tremendous excess mortality above a specific threshold temperature (Baccini et al. 2008; D’Ippoliti et al. 2010). Because governments and municipalities are under pressure to create suitable and effective adaptation and mitigation plans, there is a need to scientifically assess and evaluate different strategies. The primary objective of this study was to assess, on a small-scale intra-urban level, the influence of urban green and blue on heat-related all-cause natural excess mortality in Lisbon between 1998 and 2008. The underlying assumption was that areas with a higher share of vegetation or within closer proximity to a large water body display lower outdoor (equivalent) temperatures leading to reduced heat exposure and thus reduced heat-related mortality.

## Data and Methods

### Meteorological and Air Pollution Data

Hourly values of temperature, humidity, wind speed, and cloud coverage for one station located at the Lisbon airport were purchased from the U.S. National Climatic Data Centre (http://www.ncdc.noaa.gov/). To account for the complex effects of various meteorological parameters, we determined the universal thermal climate index (UTCI), which is a measure of equivalent temperature based on the Fiala model (Fiala et al. 2012). To determine the UTCI, the input variable, mean radiant temperature, is modeled as a function of temperature and cloud coverage using RayMan, version 1.2 (Matzarakis et al. 2007). Daily means of temperature and UTCI were calculated from hourly values.

To assess the air pollution in Lisbon, we intentionally chose urban background stations for the analysis in an attempt to cover large-scale pollution levels and day-to-day variations in exposure rather than extreme values at a highly contaminated site. For O_3_ pollution, we chose data from three urban background stations located in Beato (O_3_ data from 1998 to 2008), Hospital Velho (O_3_ data from 1998 to 2007), and Alfragide/Amadora (O_3_ data from 2001 to 2008). For PM_10_ (particulate matter with an aerodynamic diameter of < 10 μm) pollution, we chose data from stations in Olivais (PM_10_ data from 1999 to 2008), Mem-Martins (PM_10_ data from 2002 to 2008), Quinta do Marquês (PM_10_ data from 2002 to 2008), and Reboleira (PM_10_ data from 2002 to 2008). Daily means were calculated from hourly PM_10_ and O_3_ measurements, if at least 75% of the data was available for a particular day. Approximately 10% of the daily PM_10_ and 8% percent of the daily O_3_ data were missing. One city-specific spatial average was then calculated for each pollutant as an arithmetic mean from the individual stations. If data for one station was missing, the spatial mean value was calculated from the other stations. Because the time-series showed large gaps stretching over several years, we conducted a sensitivity analysis using only data from one station. The outcomes were not affected by this (data not shown) and in the final analysis, spatially averaged values were included.

### Mortality Data

**Figure 1:**
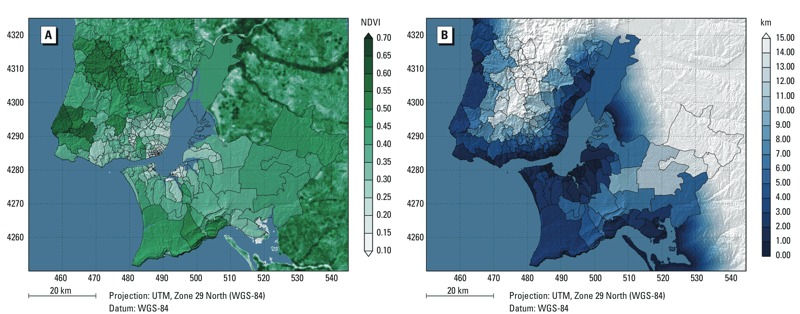
Maps of the NDVI distribution in Lisbon (*A*) per parish and mean distance to the Atlantic Ocean or Tagus Estuary (*B*).

Daily age-stratified death counts from 1998 to 2008 for each civil parish in the Lisbon Metropolitan area were provided by the Instituto Nacional de Estatística. There are 213 civil parishes (freguesia) in Lisbon (see Figure 1) and the deaths were recorded at the registered address. In our analysis, only deaths of inhabitants above the age of 65 years were included, as previous studies have demonstrated an increased risk for heat in the elderly (Basu 2009; Basu and Samet 2002). We abstained from further stratification by cause of death to retain sufficient numbers of cases for the statistical analysis. In total, 218,764 deaths between 1998 and 2008 were analyzed.

### Socioeconomic Data on Parish Level

Parish level socioeconomic data for 2001 and 2011 were obtained from the Instituto Nacional de Estatística and averaged over the 2 years to reflect the characteristics of each parish during the study period. We conducted a principal component analysis (PCA) with varimax rotation as an exploratory analysis and identified three primary components related to age, population and building density, and education (see Table S2). From the variables included in each component, we selected the percentage of persons > 65 years of age, the number of residents per km^2^, and the percentage of college graduates to represent age, density, and education, respectively, in our final models. In addition, we included the percentage receiving social benefits as a proxy for socioeconomic status (SES). For the sensitivity analysis, we evaluated alternative variables from each component group and found that the choice of variable had little or no effect on associations between temperature and mortality (data not shown).

### Remote Sensing Data

The amount and spatial distribution of urban green is quantified using a common remotely sensed vegetative index, the Normalized Difference Vegetation Index (NDVI) (Tucker 1979). The NDVI is a dimensionless index that describes the difference between the visible (VIS) and near-infrared (NIR) reflectance of vegetation canopies. The lower NDVI limit for land-surfaces (except water) is approximately zero and the upper limit is approximately 0.8 (Huete et al. 1999). The relation between NDVI and fractional vegetation is well-described in the literature (e.g., Carlson and Ripley 1997). The moderate resolution imaging spectroradiometer (MODIS) sensor aboard the Terra satellite provided data for this study. We used the global MODIS product MOD13Q1 version 005. This product is a 16-day composite that provides cloud-free, atmospherically corrected, and nadir-adjusted data of gridded NDVI values at a spatial resolution of 250 m (Huete et al. 1999).

The MODIS product MOD11A2 version 005 (Wan 2008) was used to quantify the average daytime and nighttime land surface temperature (LST). This product is a composite that uses an average of all clear-sky measurements obtained during the 8-day compositing period (Wan 2008). The spatial resolution of the LST pixel is 1 km at nadir.

Remote sensing data include all summer months (June, July, August) for the years 2000–2008 and encompass 54 NDVI images, 104 daytime LST images, and 104 nighttime LST images. The temporal coverage of the remote sensing data matches the mortality data (1998–2008) as best as possible. There are no MODIS products before 2000. The data availability varies for each image pixel depending on cloud cover. The data availability is between 97% and 99% for the daytime LST pixels and between 90% and 100% for nighttime LST pixels. The NDVI composites have no missing values.

For converting the gridded satellite products into parish-level information, we initially calculated the temporal average for each NDVI and LST pixel. We did not include temporal changes of NDVI or LST into our analysis. Then the universal transverse mercator (UTM) coordinates of each parish polygon were transformed into the coordinates of the Sinusoidal projection of the MODIS grid. The center point of each MODIS pixel is used to identify the pixels that are belonging to a specific parish. Finally we calculated the spatial mean of NDVI and LST for each parish.

The digital elevation model from the NASA Shuttle Radar Topographic Mission (SRTM; http://srtm.csi.cgiar.org) was used to generate a land-water mask for the study region. The mask has a horizontal resolution of 90 m. The distance to water was calculated for each pixel, that is, each pixel was assigned an approximate Euclidean distance to the Atlantic Ocean and Tagus Estuary. Then we calculated the mean distance for the entire parish area.

### Statistical Analysis and Modeling

***Distributed lag nonlinear models.*** We used distributed lag nonlinear models (DLNMs) to assess the time structure of associations between heat and mortality among those > 65 years of age. DLNMs use smooth nonlinear functions to simultaneously describe the shape of the relationship along both the space of a predictor variable, in our case UTCI, and the lag dimension of its occurrence (Gasparrini 2011; Gasparrini et al. 2010). The cross-basis function was included in generalized additive models (GAMs) adjusted for covariates selected using the unbiased risk estimator (UBRE), a rescaled Akaike’s information criterion, to identify the variables that provided the best model fit (Wood 2006). In addition, we excluded potential covariates that were not significant predictors of mortality (*p* > 0.05). Potential covariates included long-term and seasonal trends [using penalized splines with 4, 5, or 6 degrees of freedom (df) per year, or categorical variables for year and season]; day of the week; and daily mean, minimum, or maximum PM_10_ and O_3_ concentrations averaged over the same day and 1 previous day (lag 0–1), or over 14 days (lag 0–13) (Burkart et al. 2013). We evaluated lag periods for UTCI of 1 to 30 days, and centered UTCI at the annual median UTCI (8.3°C UTCI); the df for UTCI and lags were set to 5 in the final DLNM but effect estimates were largely unaffected by the specification of df (data not shown). Moreover, effect estimates for UTCI and mortality were largely unaffected by the choice of specific confounder covariates (data not shown), and the final DLNM model included daily mean UTCI, long-term and seasonal trends (penalized splines with 6 df per year), and average PM_10_ and O_3_ concentrations over the actual and previous day (lag 0–1). All analyses were carried out using R (version 2.11.0; R Core Team) and the package “dlnm” (Gasparrini 2011).

***Interaction modeling.*** To assess possible mitigation of the association between heat and mortality by urban green, we modeled interactions between UTCI and average NDVI during June–August. To categorize areas of urban green we used quartiles as cut-off values; each parish was assigned to a category and eventually mortality counts were aggregated over each category. As an alternative to NDVI we also evaluated interactions between heat and quartiles of mean LST during June–August, with separate models used to assess modification of associations between heat and mortality by daytime LST and nighttime LST. To assess the potential influence of urban blue, we modeled interactions between temperature and the mean distance of each parish from the Atlantic Ocean and Tagus Estuary coast. We performed exploratory analyses using distances from 1 to 10 km to dichotomize proximity to water (data not shown), and categorized proximity as ≤ 4 km or > 4 km based on the exploratory approach.

Based on the DLNM analyses, we evaluated average values of daily mean UTCI on the same day and previous 2 days (lag 0–2), and quantified the estimated heat effects by modeling UTCI as a segmented linear variable using either the 95th or 99th percentile of the UTCI in the entire study area during 1998–2008 as the threshold (19.9°C or 24.8°C, respectively), such that model estimates represent the difference in mortality with a 1°C increase in lag 0–2 UTCI above the threshold value. Separate GAMs including daily data for the entire years from 1998 to 2008 were fit using the R package “mgcv” (Wood 2006) to model interactions between heat (UTCI above the 95th or 99th percentile) and quartiles of NDVI (or LST) and proximity to water (≤ 4 km or > 4 km). Models were adjusted for time trend (penalized splines with 6 df per year), average daily mean PM_10_ and O_3_ concentrations (lag 0–1), percentage of the parish population > 65 years of age, building density (number of buildings per km^2^), percentage of college graduates, and the proportion of inhabitants receiving social benefits. The final interaction models were adjusted for trend using 6 df per year, day of the week, and air pollution using 2-day lags of PM_10_ and O_3_. All variables included were proofed to have a significant influences (*p* < 0.05). To account for population growth, population was included as an offset. In addition, we included the area (in km^2^) of each formed category (i.e., NDVI, LST, or distance to coast) as weights in each model. To assess the robustness of our models, we conducted a sensitivity analysis analogue to the DLNM analysis: We tested 4, 5, and 6 df for trend adjustment and included 2- and 14-day lags of mean, minima, and maxima PM_10_ and O_3_. Moreover, we fitted models including one and two pollutants. Model outcomes were hardly affected by these specifications, and we considered them as being robust. In addition, we fitted interaction models using daily mean temperature as predictive variable instead of UTCI and predicted the mortality increase above the 95th and 99th temperature percentile (24.0°C or 27.4°C, respectively). This was done to compare differences in outcomes and to compare the predictive power (based on the UBRE score) of both variables. Outcomes for temperature are presented in Figures S1–S6. The GAMs were fitted using the R package “mgcv” (Wood 2006).

We present formulas for the final models: Equation 1 represents the model for analyzing the influence of urban green; Equation 2 represents the model for analyzing the influence of proximity to the water:

Ln(*E_y_*) = ln(pop*_y_*) + *f*(*t*) + γ*_i_x_i_* + α*_j_w_j_* + λ*_k_*(coast*_k_*) + β_1_(NDVI) + β_2_(UTCI) + β_3_(NDVI * UTCI_[_ *_UTCI_*_-δ]_) [1]

Ln(*E_y_*) = ln(pop*_y_*) + *f*(*t*) + γ*_i_x_i_* + α*_j_w_j_* +λ*_l_*(NDVI*_l_*) + β_1_(coast) + β_2_(UTCI) + β_3_(coast * UTCI_[_ *_UTCI_*_-δ]_), [2]

where *Ey* indicates the predicted mortality in area *y*; ln(pop*_y_*) is an offset term for the population size in area *y*; *f*(*t*) indicates the smoothed time trend; γ*_i_* represents model coefficients for the *i* covariates *x_i_* (day of the week, age, density, education, and SES); α*_j_* indicates the coefficients for the *j* covariates *w_j_* (PM_10_ and O_3_); λ*_k_* represents model coefficients for the *k* distance to coast indicator variables (*k* = 1, 2); and β1, β2, and β3 represent model coefficients for NDVI and UTCI; δ indicates the breakpoint equivalent temperature, in our case the 95th and 99th UTCI percentile. The interaction models for proximity to coast (and LST) were specified accordingly with λ*_l_* representing model coefficients for the *l* NDVI indicator variables (*l* = 1, 2, 3, 4).

## Results

### Distribution of and Relationship between Environmental, Spatial and Socioeconomic Characteristics

Figure 1A depicts the study area (Lisbon Metropolitan Area) and the spatial distribution of urban green for the civil parishes. The mean NDVI is 0.34, with a minimum value of 0.13, a maximum value of 0.62 and a standard deviation of 0.10. The mean distance to the water (i.e., the Atlantic Ocean and the Tagus Estuary) is displayed in Figure 1B. The smallest mean distance for one civil parish is 0.3 km, the largest is 20.5 km. The average distance to the water is 6.3 km, with a standard deviation of 4.3.

Table 1 shows mean values for parish characteristics according to NDVI quartiles (urban green) and proximity to water (urban blue). Parishes with higher vegetation coverage generally show lower LSTs, especially during nighttime. The Spearman correlation coefficient (between parishes) for NDVI and nighttime LST is –0.75, and between NDVI and daytime LSTs is –0.34. Moreover, there is a negative correlation of –0.53 between NDVI and building density. Greener areas are further away from the water with a correlation of 0.47 between NDVI and average distance to the coast. The proportion of residents > age 65 years and NDVI show no explicit association, with a weak correlation coefficient of –0.24. Generally, the proportion of college graduates is higher in less green and more densely built areas: there is a weak negative correlation of –0.25 between NDVI and proportion of college graduates. The areas in closer proximity to the water show higher nighttime LSTs but lower daytime LSTs with a negative correlation of –0.54 between distance to water and nighttime LSTs and a positive correlation of 0.35 between distance to water and daytime LSTs. In addition, the building density is higher in areas in closer proximity to the sea, with a negative correlation of –0.37 between distance to water and building density. The proportion of the population > 65 years is higher in areas closer to the water: there is a negative correlation of –0.36 between proportion of the population > 65 years and distance to water. The proportion of college graduates is higher in areas within the 4-km zone of the water; distance to water and the proportion of college graduates correlate, with a correlation coefficient of –0.32.

**Table 1:** Socioeconomic and spatial information by NDVI and coastal proximity across 203 Lisbon parishes between 1998 and 2008 (mean ± standard deviation unless otherwise indicated).

Exposure	NDVI^*a*^	Night-time LST^*b*^ [°C]	Day-time LST^*b*^ [°C]	Distance to water^*c*^ [km]	Percentage > 65 years of age	Percentage of college graduates	Social benefits^*d*^ [/1,000]	Building density [/km^2^]	Population [*N*]	Area [km^2^]
NDVI quartile
Quartile 1 (< 0.27)	0.23 ± 0.03	18.4 ± 0.6	33.9 ± 2.0	3.7 ± 2.5	21.7 ± 6.2	27.1 ± 14.2	6.1 ± 4.4	116 ± 927	4,875	96.4
Quartile 2 (0.27–0.33)	0.29 ± 0.02	18.0 ± 0.6	33.9 ± 2.1	4.9 ± 2.7	18.6 ± 6.5	30.8 ± 17.3	4.7 ± 2.5	609 ± 396	847,820	256.8
Quartile 3 (0.34–0.41)	0.37 ± 0.02	17.2 ± 0.8	33.6 ± 2.8	6.7 ± 3.6	15.4 ± 4.0	20.3 ± 11.2	5.4 ± 5.4	318 ± 198	923,743	810.9
Quartile 4 (> 0.41)	0.48 ± 0.05	16.5 ± 1.2	31.8 ± 2.9	8.1 ± 4.6	17.9 ± 4.9	20.1 ± 9.96	3.6 ± 2.8	203 ± 271	384,322	1558.3
Proximity to water
≥ 4 km	0.37 ± 0.09	11.9 ± 1.0	24.0 ± 1.2	8.6 ± 3.4	16.5 ± 5.5	20.0	4.7 ± 4.2	348 ± 282	1,782,117	2273.5
< 4 km	0.32 ± 0.09	12.8 ± 0.8	22.7 ± 1.6	2.5 ± 1.0	21.0 ± 5.4	29.8	5.4 ± 3.8	899 ± 832	958,670	661.2
^***a***^Mean NDVI for each parish in June, July, and August during 2000–2008. ^***b***^Mean daytime/nighttime LST for each parish in June, July, and August during 2000–2008. ^***c***^Mean distance of each parish to the Atlantic Ocean or the Tagus Estuary. ^***d***^Number of parish residents receiving social security benefits per 1,000.										

### Lag Structure of Heat Effects

**Figure 2:**
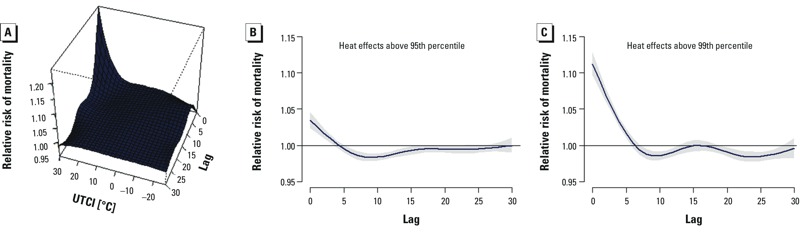
Three-dimensional DLNM outputs of the relative risk of mortality along equivalent temperature and lags for all-cause mortality (*A*) and plots of relative risk of mortality at the 95th (*B*) and 99th (*C*) percentiles of equivalent temperature distribution (corresponding to a UTCI of 19.9°C and 24.8°C, respectively), with reference at the median equivalent temperature (8.3°C UTCI) where the relative risk equals 1 for the elderly above the age of 65 years. Outputs are adjusted for long-term and seasonal trend (6 df per year), daily averages of O_3_ and PM_10_ (lags 0–1). Grey areas represent the upper and lower 95% confidence intervals.

Figure 2A represents a three dimensional DLNM output of the exposure lag–response relationship for mortality in individuals > 65 years old. The results demonstrate higher mortality with higher UTCI and shorter lag periods. To show the temporal relation between UTCI and mortality more clearly, we show relative risks for UTCI above the 95th and 99th percentiles (19.9°C and 24.8°C, respectively) compared with the median value of UTCI (8.3°C) according to number of lag days (Figure 2B,C). The strongest associations are with UTCI on the same day, followed by positive but decreasing relative risks (RR) for several days. Subsequent RRs < 1 suggest harvesting, a phenomenon that results from a short-term forward shift in the death rate of frail individuals (Gasparrini et al. 2010; Gasparrini 2011).

### Heat Effects by Vegetation Coverage and Coastal Proximity

**Figure 3:**
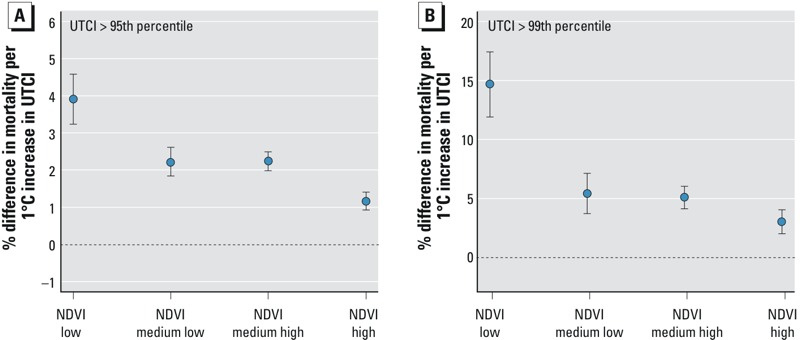
Difference in mortality increase among those > 65 years of age with a 1°C increase in UTCI above the 95th (*A*) and 99th (*B*) percentiles lags 0–2 for NDVI quartiles with 95% confidence intervals. GAMs allowing for interaction between UTCI and different NDVI classes were adjusted for long-term and seasonal trend (6 df per year), daily averages of O_3_ and PM_10_ (lag 0–1), percent of parish population > 65 years of age, building density, proportion of college graduates, and percentage of population receiving social benefits.

The association between mortality among those > 65 years of age and a 1°C increase in UTCI above the 95th percentile (19.9°C) was strongest for parishes in the lowest quartile of NDVI, with an estimated increase in mortality of 3.9% (95% CI: 3.3, 4.6%), in contrast with 2.2% (95% CI: 1.8, 2.6%), 2.2% (95% CI: 2.0, 2.5%), and 1.2% (95% CI: 0.9, 1.4%) for the 2nd, 3rd, and 4th quartiles of NDVI (indicating higher levels of vegetation), respectively (Figure 3A). Mortality in association with a 1°C increase in UTCI above the 99th percentile (24.8°C) showed a similar pattern from the lowest to highest NDVI quartile [14.7% (95% CI: 11.9, 17.5%), 5.4% (95% CI: 3.7, 7.1%), 5.1% (95% CI: 4.1, 6.0%), and 3.0% (95% CI: 2.0, 4.0%), respectively] (Figure 3B).

**Figure 4:**
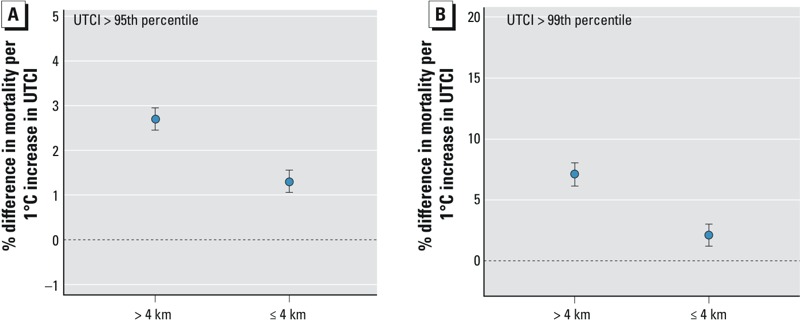
Difference in mortality increase among those > 65 years of age with a 1°C increase in UTCI above the 95th (*A*) and 99th (*B*) percentile for lags 0–2 for areas with a distance of > 4 km and ≤ 4 km to the Atlantic Ocean and Tagus Estuary with 95% confidence intervals. GAMs allowing for interaction between UTCI and distance classes were adjusted for long-term and seasonal trend (6 df per year), daily averages of O_3_ and PM_10_, percent of parish population > 65 years of age, building density, proportion of college graduates, and percentage of population receiving social benefits.

The association between mortality and a 1°C increase in UTCI above the 95th percentile was stronger for parishes > 4 km from the Atlantic Ocean or Tagus Estuary (2.7% higher mortality; 95% CI: 2.4, 3.0%) than parishes ≤ 4 km from water (1.3%; 95% CI: 1.0, 1.6%) (Figure 4A). For UTCI above the 99th UTCI percentile, the corresponding estimates were 7.1% (95% CI: 6.2, 8.1%) and 2.1% (95% CI: 1.2, 3.0%), respectively (Figure 4B).

Associations between mortality and UCTI above the 95th and 99th percentiles also increased from the lowest to highest quartile of nighttime LST (see Figure S1). Although associations between mortality and UTCI also were weakest for the lowest quartile of daytime LST, associations did not increase monotonically with higher quartiles of daytime LST (see Figure S2). Model outputs for air temperature are included in Figures S3–S6. Conclusions are the same as drawn for UTCI. Moreover, judging by the UBRE score, there was no difference in the predictive power of both variables.

## Discussion

This study highlights the relevance of urban green and urban blue in heat effect mitigation in Lisbon. Associations between heat and mortality among the elderly were substantially weaker in parishes with higher vegetation cover and in closer proximity to the Atlantic Ocean or Tagus Estuary, consistent with mitigation of the effects of heat on mortality by urban vegetation and urban water.

Although several studies have demonstrated differences in the heat-health relationship between different cities or population groups, studies on intra-urban differences are still limited. Analyzing data from the city of Berlin and the federal state of Brandenburg, Gabriel and Endlicher (2011) found that, during the heat waves in 1994 and 2006, Berlin, and in particular its most densely built-up districts, had the highest mortality rates. Similarly, Schuster et al. (2014) found spatial heterogeneity in heat mortality in Berlin. Smoyer et al. (2000) showed that cities with the greatest heat-related mortality in Southern Ontario, Canada, had relatively high levels of urbanization and high costs of living. The French Observatoire régional de santé d’Ile-de-France (ORSIF 2003) noted that deaths during the 2003 heat wave were concentrated in poorer neighborhoods with higher levels of immigrants and substandard housing. Klein Rosenthal et al. (2014) found significantly increased mortality rates in New York among the elderly during hot days in neighborhoods with high poverty rates, poor housing conditions, lower rates of access to air-conditioning, impervious land cover and high surface temperatures. Similarly, high-risk areas were associated with a lack of high-income earners and higher population density in Brisbane, Australia (Hondula and Barnett 2014). In Philadelphia heat mortality neighborhoods were more likely to have low housing values and a higher proportion of Black residents (Uejio et al. 2011) and Gronlund et al. (2015) found that in Michigan green space, housing, and social isolation may independently enhance elderly peoples’ heat-related cardiovascular mortality vulnerability. In another study analyzing data from Barcelona, Xu et al. (2013) found that associations between heat and mortality were stronger among residents who perceived low levels of surrounding greenness.

In this study we explicitly focused on the effects of urban green and blue whilst adjusting for other confounders. One central issue in this type of research is the relationship between urban vegetation, surface, and air temperatures. As outlined in the introduction, vegetation cools the urban atmosphere through shading and evapotranspiration but might have a negative effect on thermal comfort in summer by reducing wind speed. In a study investigating the urban local-scale climate in Lisbon, Alcoforado et al. (2014) showed that higher UHI intensities occur during nighttime. Generally, the UHI is a nighttime phenomenon: the urban environment heats up moderately during early hours of the day due to the high heat capacity and heat conductivity of urban artificial materials. During nighttime, the stored heat is released gradually resulting in a slower cooling of urban areas, whereas the natural rural surroundings cool down comparably fast. Accordingly, greener areas in the city mostly heat up rather quickly but cool down similarly quickly during nighttime. Our finding of a weaker correlation between NDVI and daytime LST (–0.34) compared with nighttime LST (–0.75) is thus consistent with expectations.

We observed a strong correlation between NDVI and nighttime LST and also found diminished heat effects in areas with lower nighttime LSTs. However, surface temperatures might not be a good proxy for actual air temperatures. Due to shading in densely built-up areas and street canyons, ground surface temperatures might be substantially lower than rooftops. Moreover, the advection of air masses could potentially interfere with the surface-air temperature relationship. Moreover, ambient UTCI as measure of heat exposure may not have fully captured the actual exposures of the populations, which is also influenced by factors such as time spent indoors, building type, floor level, access to air conditioning, and behavioral aspects (schedules, preferences, lifestyle, etc.) (cf., Kuras et al. 2015).

A strength of this study lies in the local scale intra-urban analysis of heat-related risk and the investigation of the mitigation potential of urban green and blue on heat effects. Nonetheless, there are certain limitations which we would like to acknowledge. Although we carefully attempted to account for potential confounders (e.g., age, urban density, socioeconomic status) residual confounding cannot be fully ruled out. Using NDVI as a proxy for urban green is a practical and feasible approach as it can easily be transferred to other cities and thus enables the comparison of studies and study areas. Nonetheless, the NDVI does not provide any qualitative information about the type of vegetation (e.g., shade tree, lawn), its shape and height or the type of use (e.g., alley, park, cemetery). Furthermore, the vegetation might influence air pollution levels. Nowak et al. (1998) argue that reduced air temperature due to trees can improve the air quality because the emission of many pollutants and/or precursor chemicals are temperature-dependent. Although we adjusted our models for PM_10_ and O_3_, we might not have been able to sufficiently consider the interaction effect between (equivalent) temperature and air pollutants that has been demonstrated in a previous study (Burkart et al. 2013). The observed decrease in mortality in green areas might be partly due to a reduced air pollution level. Similarly, our analysis allows no definite conclusion regarding whether the effect of urban green is due to reduced temperatures or whether at least parts of the mitigation effect are due to an improved health status of the population living in the green areas. Similarly, it is unclear whether the mitigating effect of urban green is indeed due to reduced temperatures or if urban vegetation possibly exerts an indirect effect by reducing stress thus increasing coping capacities of residents. This study has analyzed general heat effects above a threshold but has not looked at heat episodes occurring over an extended period of time. Under such conditions, the vegetation might lose its cooling effect as vegetation might dry out. Another limitation in the assessment of urban blue should be considered. In our analysis of water proximity we did not distinguish between the Atlantic Ocean and the Tagus Estuary. Because the Atlantic water is much cooler than the estuary water, the cooling effect of the Atlantic is likely to be greater. To maintain sufficient statistical power, we did not differentiate between the two water sources in our models. Nonetheless, if a larger data set is used, such stratifications might be worth considering in further research.

In this study, UTCI and temperature were equally strong predictors of mortality. Similarly, other authors could not find a predictive advantage of thermal indices such as heat index or perceived equivalent temperature (Barnett et al. 2010; Burkart et al. 2011; Zhang et al. 2012). While this initially seems surprising given the theoretical advantage of the UTCI or other equivalent temperatures, it needs to be considered that a great share of the variance in equivalent temperatures is usually explained by the variance in temperature. Nonetheless, there might be extreme situations when temperature does not sufficiently reflect thermal conditions. Particularly, during periods of very high temperatures, elevated humidity, and/or no air movement might crucially affect the thermal environment and thus heat-related excess mortality. This study design might not have been suitable to capture such extreme situations, and we suggest further research on the role of other meteorological parameters, apart from temperature, during heat waves. Another crucial question this study brought up is on harvesting. We found that above the 95th percentile, harvesting effects almost compensated for heat excess mortality as the RR at higher lags dropped below the expected value, whilst above the 99th percentile we observed heat effects that did not constitute a bringing forward of deaths in time; here the drop in RR after several days did not compensate for the preceding increase.

The findings of this study call for efforts and practical implementations of blue and green spaces. Following joint work with the Municipality of Lisbon (from 2005 onwards) and the publication of a paper on climatic principles to urban planning (Alcoforado et al. 2009), the Municipality has included indications regarding the importance of keeping free paths to the Tagus breezes and of creating and/or preserving existing green areas. Nonetheless, such implementations are challenging and generally there are tradeoffs between ecological benefits and disadvantages. Water demand and availability are especially problematic given the Mediterranean type climate, which is dry in the hot season. In some gardens and parks, native plants are chosen, but their effect in increasing air humidity and thus reducing air temperature (through evapotranspiration) is limited, as their strategy is to avoid water loss. Nevertheless, some studies highlight the relevance of shading rather than evapotranspiration (Shashua-Bar et al. 2009; Gross 2012; Saneinejad et al. 2014). Hence, sensible and effective installation of urban green and blue infrastructure calls for collaboration between urban planners, climatologists, botanists, and public health experts.

## Conclusion

Urban vegetation and proximity to water both were associated with decreased heat-related mortality in the metropolitan area of Lisbon. Specifically, associations between mortality and 1°C increases in UTCI above the 95th and 99th percentiles were substantially reduced in parishes with high NDVI and in parishes in closer proximity to the Atlantic Ocean and Tagus Estuary after adjusting for time trends, and for indicators of the age, density, and socioeconomic status of the populations in each parish. Our findings should help inform town planners and decision makers involved in developing climate change mitigation and adaptation strategies, particularly in southern European cities that may be especially vulnerable to adverse heat effects.

## Supplemental Material

(822 KB) PDF

## References

[r1] Alcoforado MJ, Andrade H (2006). Nocturnal urban heat island in Lisbon (Portugal): main features and modelling attempts.. Theor Appl Climatol.

[r2] Alcoforado MJ, Andrade H, Lopes A, Vasconcelos J (2009). Application of climatic guidelines to urban planning: the example of Lisbon (Portugal).. Landsc Urban Plan.

[r3] Alcoforado MJ, Lopes A, Alves E, Canário P (2014). Lisbon heat island: statistical study (2004–2012).. Finisterra.

[r4] Andrade H, Alcoforado MJ (2008). Microclimatic variation of thermal comfort in a district of Lisbon (Telheiras) at night.. Theor Appl Climatol.

[r5] Andrade H, Vieira R (2007). A climatic study of an urban green space: the Gulbenkian Park in Lisbon (Portugal).. Finisterra.

[r6] Baccini M, Biggeri A, Accetta G, Kosatsky T, Katsouyanni K, Analitis A (2008). Heat effects on mortality in 15 European cities.. Epidemiology.

[r7] Barnett AG, Tong S, Clements AC (2010). What measure of temperature is the best predictor of mortality?. Environ Res.

[r8] Basu R 2009 High ambient temperature and mortality: a review of epidemiologic studies from 2001 to 2008. Environ Health 8 1 40, doi:10.1186/1476-069X-8-40 19758453 PMC2759912

[r9] Basu R, Samet JM (2002). Relation between elevated ambient temperature and mortality: a review of the epidemiologic evidence.. Epidemiol Rev.

[r10] Bernatzky A (1982). The contribution of tress and green spaces to a town climate.. Energy Build.

[r11] Burkart K, Breitner S, Schneider A, Khan MM, Krämer A, Endlicher W (2014). An analysis of heat effects in different subpopulations of Bangladesh.. Int J Biometeorol.

[r12] Burkart K, Canário P, Breitner S, Schneider A, Scherber K, Andrade H (2013). Interactive short-term effects of equivalent temperature and air pollution on human mortality in Berlin and Lisbon.. Environ Pollut.

[r13] Burkart K, Schneider A, Breitner S, Khan MH, Krämer A, Endlicher W (2011). The effect of atmospheric thermal conditions and urban thermal pollution on all-cause and cardiovascular mortality in Bangladesh.. Environ Pollut.

[r14] Carlson TN, Ripley DA (1997). On the relation between NDVI, fractional vegetation cover, and leaf area index.. Remote Sens Environ.

[r15] Clarke JF (1972). Some effects of the urban structure on heat mortality.. Environ Res.

[r16] D’Ippoliti D Michelozzi P Marino C de’Donato F Menne B Katsouyanni K 2010 The impact of heat waves on mortality in 9 European cities: results from the EuroHEAT project. Environmental Health Environ Health 9 37, doi:10.1186/1476-069X-9-37 PMC2914717 20637065

[r17] Endlicher W (2012). Einführung in die Stadtökologie: Grundzüge des urbanen Mensch-Umwelt-Systems [in German]..

[r18] Fiala D, Havenith G, Bröde P, Kampmann B, Jendritzky G (2012). UTCI-Fiala multi-node model of human heat transfer and temperature regulation.. Int J Biometeorol.

[r19] Gabriel KM, Endlicher WR (2011). Urban and rural mortality rates during heat waves in Berlin and Brandenburg, Germany.. Environ Pollut.

[r20] Gasparrini A (2011). Distributed lag linear and non-linear models in R: the package dlnm.. J Stat Softw.

[r21] Gasparrini A, Armstrong B, Kenward MG (2010). Distributed lag non-linear models.. Stat Med.

[r22] Georgi JN, Dimitriou D (2010). The contribution of urban green spaces to the improvement of environment in cities: case study of Chania, Greece.. Build Environ.

[r23] Gronlund CJ, Berrocal VJ, Veronica JL, White-Newsome JL, Conlon KC, O’Neill MS (2015). Vulnerability to extreme heat by sociodemographic characteristics and area green space among the elderly in Michigan, 1990-2007.. Environ Res.

[r24] Gross G (2012). Effects of different vegetation on temperature in an urban building environment. Micro-scale numerical experiments.. Meteorologische Zeitschrift.

[r25] Häckel H (2008). Meteorologie [in German]..

[r26] Hondula DM Barnett AG 2014 Heat-related morbidity in Brisbane, Australia: spatial variation and area-level predictors. Environ Health Perspect 122 831 836, doi:10.1289/ehp.1307496 24787277 PMC4123028

[r27] Huang YJ, Akbari H, Taha H, Rosenfeld AH (1987). The potential of vegetation in reducing summer cooling loads in residential buildings.. J Clim Appl Meteor.

[r28] Huete A, Justice C, van Leeuwen W (1999). MODIS Vegetation Index (MOD13): Algorithm Theoretical Basis Document ATBD. Version 3.0.. http://modis.gsfc.nasa.gov/data/atbd/atbd_mod13.pdf.

[r29] Klein Rosenthal J, Kinney PL, Metzger KB (2014). Intra-urban vulnerability to heat-related mortality in New York City, 1997–2006.. Health Place.

[r30] Kuras ER, Hondula DM, Brown-Saracino J (2015). Heterogeneity in individually experienced temperatures (IETs) within an urban neighborhood: insights from a new approach to measuring heat exposure.. Int J Biometeorol.

[r31] Kuttler W (1998). Stadtklima.. In: Stadtökologie: Ein Fachbuch für Studium und Praxis [in German], (Sukopp H, Wittig R, eds). 2nd ed.

[r32] Kuttler W (2004). Stadtklima [in German].. UWSF–Z Umweltchem Ökotox.

[r33] Landsberg HE (1981). The Urban Climate..

[r34] Lopes A Alves E Alcoforado MJ Machete R 2013 Lisbon urban heat island updated: new highlights about the relationships between thermal patterns and wind regimes. Advances in Meteorology 2013:487695, doi:10.1155/2013/487695

[r35] Matzarakis A, Rutz F, Mayer H (2007). Modelling radiation fluxes in simple and complex environments—application of the RayMan model.. Int J Biometeorol.

[r36] Nowak DJ, McHale PJ, Ibarra M, Crane D, Stevens JC, Luley CJ (1998). Modeling the effects of urban vegetation on air pollution.. In: Air Pollution Modeling and Its Application XII, Vol. 22 (Gryning SE, Chaumerliac N, eds).

[r37] ORSIF (Observatoire régional de santé d’Ile-de-France) (2003). Conséquences sanitaires de la canicule d’août 2003 en Ile-de-France [in French].. Revue d Épidémiologie et de Santé Publique.

[r38] Oke TR (1973). City size and the urban heat island.. Atmos Environ (1967).

[r39] Oke TR (1987). Boundary Layer Climates..

[r40] Oliveira S, Andrade H, Vaz T (2011). The cooling effect of green spaces as a contribution to the mitigation of urban heat: a case study in Lisbon.. Build Environ.

[r41] Papanastasiou D, Melas D, Bartzanas T, Kittas C (2010). Temperature, comfort and pollution levels during heat waves and the role of sea breeze.. Int J Biometeorol.

[r42] Saaroni H, Ben-Dor E, Bitan A, Potchter O (2000). Spatial distribution and microscale characteristics of the urban heat island in Tel-Aviv, Israel.. Landsc Urban Plan.

[r43] Saneinejad S, Moonen P, Carmeliet J (2014). Comparative assessment of various heat island mitigation measures.. Build Environ.

[r44] Santamouris M (2001). The role of green spaces.. In: Energy and Climate in the Urban Built Environment (Santamouris M, ed).

[r45] Schönwiese CD (2008). Klimatologie [in German]..

[r46] Schuman SH (1972). Patterns of urban heat-wave deaths and implications for prevention: data from New York and St. Louis during July, 1966.. Environ Res.

[r47] Schuster C, Burkart K, Lakes T (2014). Heat mortality in Berlin – spatial variability at the neighborhood scale.. Urban Climate.

[r48] Shashua-Bar L, Pearlmutter D, Erell E (2009). The cooling efficiency of urban landscape strategies in a hot dry climate.. Landsc Urban Plan.

[r49] Smargiassi A, Goldberg MS, Plante C, Fournier M, Baudouin Y, Kosatsky T (2009). Variation of daily warm season mortality as a function of micro-urban heat islands.. J Epidemiol Community Health.

[r50] Smoyer KE, Rainham DGC, Hewko JN (2000). Heat-stress-related mortality in five cities in Southern Ontario: 1980–1996.. Int J Biometeorol.

[r51] Souch CA, Souch C (1993). The effect of trees on summertime below canopy urban climates: a case study Bloomington, Indiana.. J Arboriculture.

[r52] Streutker DR (2003). Satellite-measured growth of the urban heat island of Houston, Texas.. Remote Sensing of Environment.

[r53] Tan J, Zheng Y, Tang X, Guo C, Li L, Song G, Zhen X, Yuan D, Kalkstein AJ, Furong L, Chen H (2010). The urban heat island and its impact on heat waves and human health in Shanghai.. Int J Biometeorol.

[r54] Tucker CJ (1979). Red and photographic infrared linear combinations for monitoring vegetation.. Remote Sensing of Environment.

[r55] Uejio CK, Wilhelmi OV, Golden JS, Mills DM, Gulino SP, Samenow JP (2011). Intra-urban societal vulnerability to extreme heat: the role of heat exposure and the built environment, socioeconomics, and neighborhood stability.. Health Place.

[r56] Unger J (2004). Intra-urban relationship between surface geometry and urban heat island: review and new approach.. Climate Research.

[r57] Vasconcelos J, Lopes A (2006). Recent urban development trends and its implication on the estuarine breezes in Lisbon, Portugal.. In: Proceedings of the 6th International Conference on Urban Climate, 12–16 June 2006, Göteborg, Sweden. Göteborg, Sweden.

[r58] Wan Z (2008). New refinements and validation of the MODIS Land-Surface Temperature/Emissivity products.. Remote Sensing of Environment.

[r59] Weischet W, Endlicher W (2012). Einführung in die Allgemeine Klimatologie [in German]..

[r60] Wood SN (2006). Generalized Additive Models: An Introduction with R..

[r61] Xu Y, Dadvand P, Barrera-Gómez J, Sartini C, Marí-Dell’Olmo M, Borrell C (2013). Differences on the effect of heat waves on mortality by sociodemographic and urban landscape characteristics.. J Epidemiol Community Health.

[r62] Zhang K, Rood RB, Michailidis G, Oswald EM, Schwartz JD, Zanobetti A (2012). Comparing exposure metrics for classifying ‘dangerous heat’ in heat wave and health warning systems.. Environ Int.

