# Supplementary material for: Modification of Heat-Related Mortality in an Elderly Urban Population by Vegetation (Urban Green) and Proximity to Water (Urban Blue): Evidence from Lisbon, Portugal
Source: Environ Health Perspect. 2015 Nov 13;124(7):927–34. doi: 10.1289/ehp.1409529 (PMC4937850; doi:10.1289/ehp.1409529)
Supplement: (822 KB) PDF [file ehp.1409529.s001.acco.pdf]

Environ Health Perspect
DOI: 10.1289/ehp.1409529

Note to readers with disabilities: *EHP* strives to ensure that all journal content is accessible to all readers. However, some figures and Supplemental Material published in *EHP* articles may not conform to 508 standards due to the complexity of the information being presented. If you need assistance accessing journal content, please contact ehp508@niehs.nih.gov. Our staff will work with you to assess and meet your accessibility needs within 3 working days.

Supplemental Material

Modification of Heat-Related Mortality in an Elderly Urban Population by Vegetation (Urban Green) and Proximity to Water (Urban Blue): Evidence from Lisbon, Portugal

Katrin Burkart, Fred Meier, Alexandra Schneider, Susanne Breitner, Paulo Canário, Maria João Alcoforado, Dieter Scherer, and Wilfried Endlicher

Table of Contents

Table S1: Average values of (equivalent) temperature, air pollution and death counts for the elderly above the age of 65 years

Table S2: Socioeconomic and sociospatial information on parish level

Figure S1: Difference in mortality increase among those > 65 years of age with a 1°C increase in UTCI above the 95th (a) and 99th (b) percentiles lags 0-2 for nighttime LST quartiles with 95% confidence intervals. GAMs allowing for interaction between UTCI and different nighttime LST classes were adjusted for long-term and seasonal trend (6 df per year), daily averages of O_3 and PM_{10} (lag 0-1), percent of parish population >65 years of age, building density, proportion of college graduates and percentage of population receiving social benefits. Nighttime LST quartiles were 16.6°C, 17.7°C and 18.1°C.

Figure S2: Difference in mortality increase among those > 65 years of age with a 1°C increase in UTCI above the 95th (a) and 99th (b) percentiles lags 0-2 for daytime LST quartiles with 95% confidence intervals. GAMs allowing for interaction between UTCI and different daytime LST classes were adjusted for long-term and seasonal trend (6 df per year), daily averages of O_3 and PM_{10} (lag 0-1), percent of parish population >65 years of age,

building density, proportion of college graduates and percentage of population receiving social benefits. Daytime LST quartiles were 32.3°C, 33.8°C and 35.0°C.

Figure S3: Difference in mortality increase among those > 65 years of age with a 1°C increase in temperature above the 95th (a) and 99th (b) percentiles lags 0-2 for NDVI quartiles with 95% confidence intervals. GAMs allowing for interaction between temperature and different NDVI classes were adjusted for long-term and seasonal trend (6 df per year), daily averages of O_3 and PM_{10} (lag 0-1), percent of parish population >65 years of age, building density, proportion of college graduates and percentage of population receiving social benefits.

Figure S4: Difference in mortality increase among those > 65 years of age with a 1°C increase in temperature above the 95th (a) and 99th (b) percentile for lags 0-2 for areas with a distance of > 4 km and ≤ 4 km to the Atlantic Ocean and Tagus Estuary with 95% confidence intervals. GAMs allowing for interaction between temperature and distance classes were adjusted for long-term and seasonal trend (6 df per year), daily averages of O3 and PM10, percent of parish population >65 years of age, building density, proportion of college graduates and percentage of population receiving social benefits.

Figure S5: Difference in mortality increase among those > 65 years of age with a 1°C increase in UTCI above the 95th (a) and 99th (b) percentiles lags 0-2 for nightime LST quartiles with 95% confidence intervals. GAMs allowing for interaction between UTCI and different nighttime LST classes were adjusted for long-term and seasonal trend (6 df per year), daily averages of O_3 and PM_{10} (lag 0-1), percent of parish population >65 years of age, building density, proportion of college graduates and percentage of population receiving social benefits. Nighttime LST quartiles were 16.6°C, 17.7°C and 18.1°C.

Figure S6: Difference in mortality increase among those > 65 years of age with a 1°C increase in UTCI above the 95th (a) and 99th (b) percentiles lags 0-2 for daytime LST quartiles with 95% confidence intervals. GAMs allowing for interaction between UTCI and different daytime LST classes were adjusted for long-term and seasonal trend (6 df per year), daily averages of O_3 and PM_{10} (lag 0-1), percent of parish population >65 years of age, building density, proportion of college graduates and percentage of population receiving social benefits. Daytime LST quartiles were 32.3°C, 33.8°C and 35.0°C.

Table S1: Average values of (equivalent) temperature, air pollution and death counts for the elderly above the age of 65 years

	Temperature [a] [°C]	UTCI [a] [°C]	PM_{10} [a] [µg/m³]	O_3 [a] [µg/m³]	Death counts [b]
Average	16.5	7.8	44.8	28.5	1657
Maximum	22.6	16.9	58.8	34.9	2167
Minimum	10.9	-0.8	27.5	23.6	1356

[a]Annual average

[b]Annual monthly average

Table S2: Socioeconomic and sociospatial information on parish level

PCA	Variable	Description
DENSITY	Percentage of built-up area	Percentage (area occupied by buildings/total area) in civil parishes
	Population vs. percentage of built-up area	Civil parish population/percentage of built-up area
	Building density	Number of buildings per km²
	Proportion of foreigners	Percentage of population without portuguese nationality
	Population density	Inhabitants per km²
	Population density in buildings	Inhabitants per building
AGE	Elderly dependece index	Population aged 65 years or more as a percentage of the population between 15 and 64 years.
	Percentage of population aged 65 years and above	Population ages 65 and above as a percentage of the total population
	Aging buildings index	Number of buildings (Buildings built until 1945 / Buildings built after 1991) * 100
	Average age of buildings (years)	[(Number of buildings aged in class j * middle point of class j)/ Total buildings]*100
	Average age of residents (years)	Sum of ages of the resident population/ Resident population
	Widowers	Percentage of widowers
EDUCATION	Population with compulsory education	Percentage of population that completes compulsory education
	Population with an academic graduation	Percentage of population with college degree
	More socially valued professions	(Employed population in most valued professions / employed population)*100
	Illiteracy rate	[(Resident population 10 years and over (can not read or write) / resident population 10 years and over]*100
not assigned	Sex ratio	Ratio of the population numbers of female and male
	Sex ratio > 65 yrs.	Ratio of the population numbers of female and male (>65 years)
	Distance to nearest hospital	Km
	Institutionalized population (total)	Percentage of population living in all kind of institutions
	Institutionalized people (in social suport and health institutions)	Percentage of population living only in social suport and health institutions
	Social security benefits /10000 inhab.	Population living from social security benefits /10000 inhabitants

Source: Portuguese Instituto Nacional de Estatística

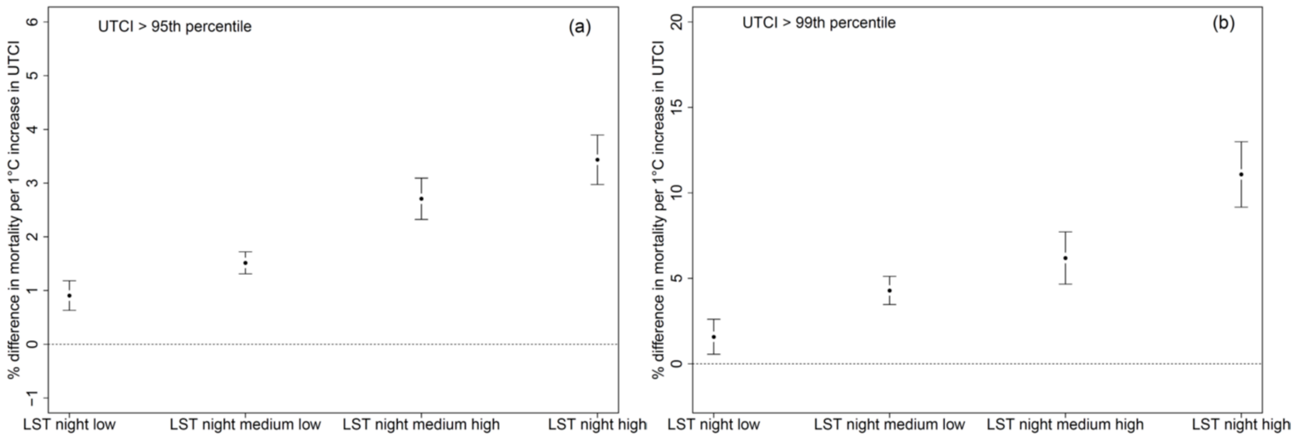

Figure S1: Difference in mortality increase among those > 65 years of age with a 1°C increase in UTCI above the 95th (a) and 99th (b) percentiles lags 0-2 for nighttime LST quartiles with 95% confidence intervals. GAMs allowing for interaction between UTCI and different nighttime LST classes were adjusted for long-term and seasonal trend (6 df per year), daily averages of O_3 and PM_{10} (lag 0-1), percent of parish population >65 years of age, building density, proportion of college graduates and percentage of population receiving social benefits. Nighttime LST quartiles were 16.6°C, 17.7°C and 18.1°C.

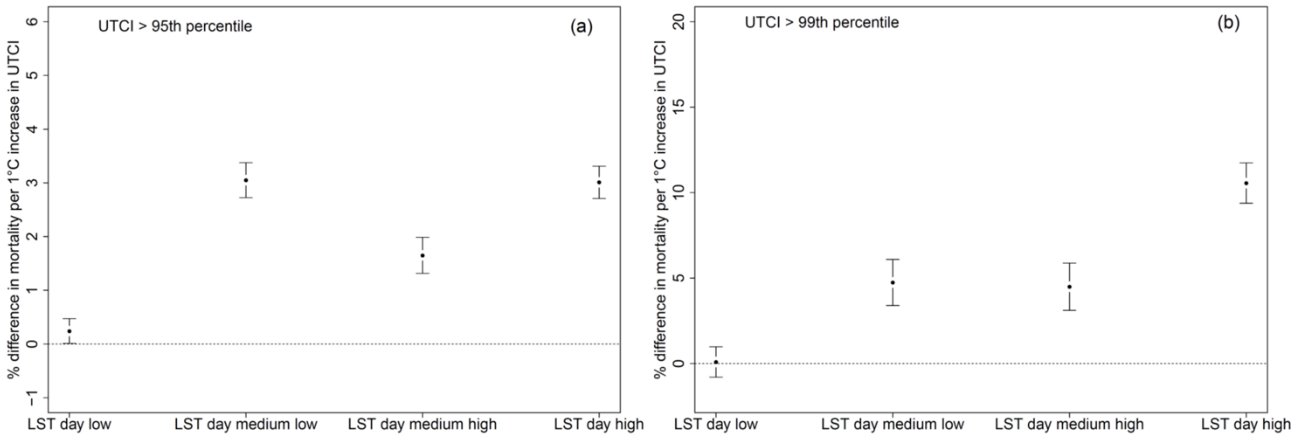

Figure S2: Difference in mortality increase among those > 65 years of age with a 1°C increase in UTCI above the 95th (a) and 99th (b) percentiles lags 0-2 for daytime LST quartiles with 95% confidence intervals. GAMs allowing for interaction between UTCI and different daytime LST classes were adjusted for long-term and seasonal trend (6 df per year), daily averages of O_3 and PM_{10} (lag 0-1), percent of parish population >65 years of age, building density, proportion of college graduates and percentage of population receiving social benefits. Daytime LST quartiles were 32.3°C, 33.8°C and 35.0°C.

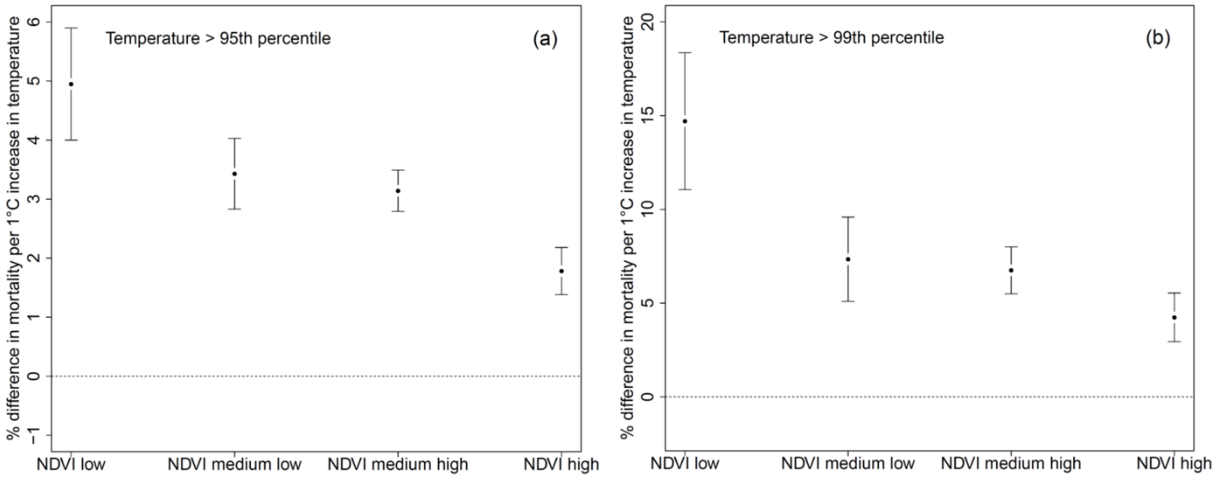

Figure S3: Difference in mortality increase among those > 65 years of age with a 1°C increase in temperature above the 95th (a) and 99th (b) percentiles lags 0-2 for NDVI quartiles with 95% confidence intervals. GAMs allowing for interaction between temperature and different NDVI classes were adjusted for long-term and seasonal trend (6 df per year), daily averages of O_3 and PM_{10} (lag 0-1), percent of parish population >65 years of age, building density, proportion of college graduates and percentage of population receiving social benefits.

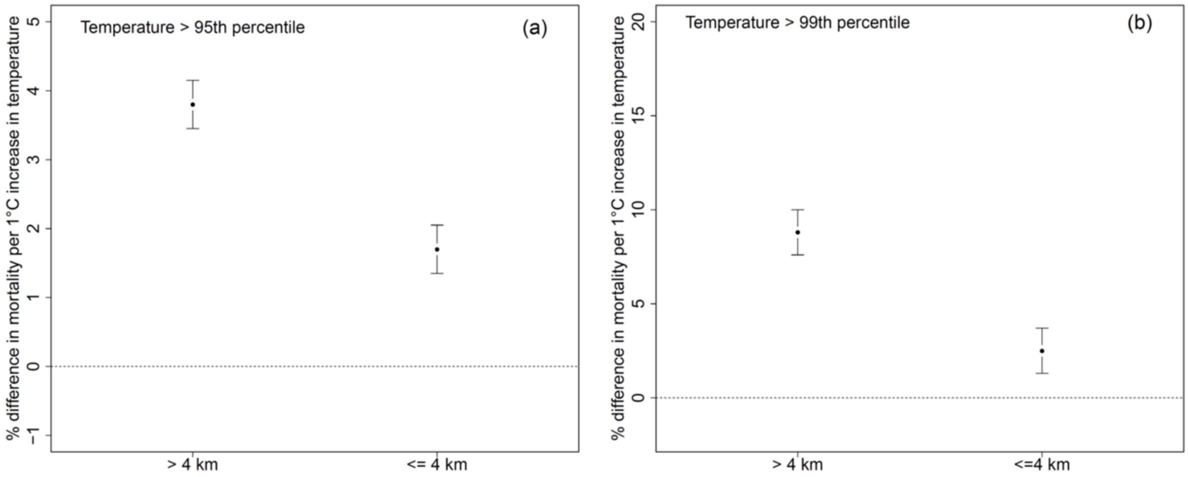

Figure S4: Difference in mortality increase among those > 65 years of age with a 1°C increase in temperature above the 95th (a) and 99th (b) percentile for lags 0-2 for areas with a distance of > 4 km and ≤ 4 km to the Atlantic Ocean and Tagus Estuary with 95% confidence intervals. GAMs allowing for interaction between temperature and distance classes were adjusted for long-term and seasonal trend (6 df per year), daily averages of O3 and PM10, percent of parish population >65 years of age, building density, proportion of college graduates and percentage of population receiving social benefits.

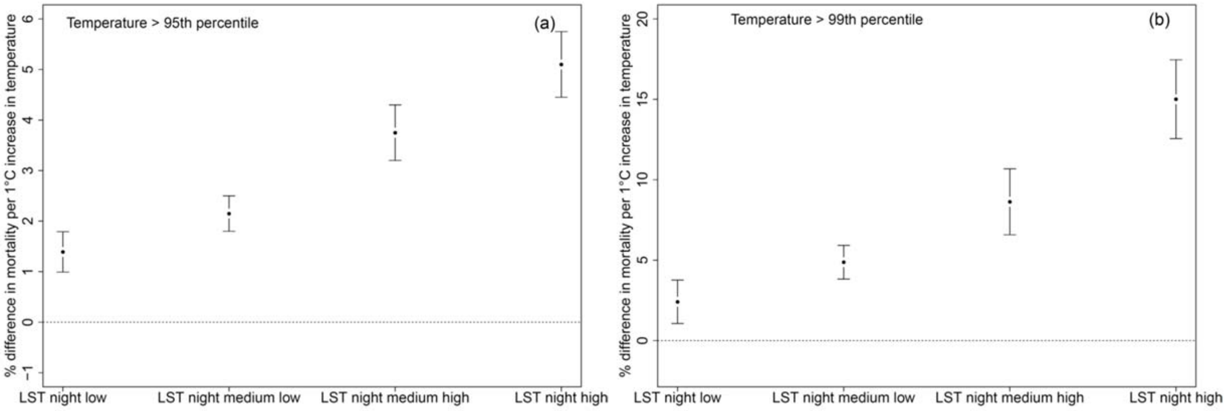

Figure S5: Difference in mortality increase among those > 65 years of age with a 1°C increase in UTCI above the 95th (a) and 99th (b) percentiles lags 0-2 for nightime LST quartiles with 95% confidence intervals. GAMs allowing for interaction between UTCI and different nighttime LST classes were adjusted for long-term and seasonal trend (6 df per year), daily averages of O_3 and PM_{10} (lag 0-1), percent of parish population >65 years of age, building density, proportion of college graduates and percentage of population receiving social benefits. Nighttime LST quartiles were 16.6°C, 17.7°C and 18.1°C.

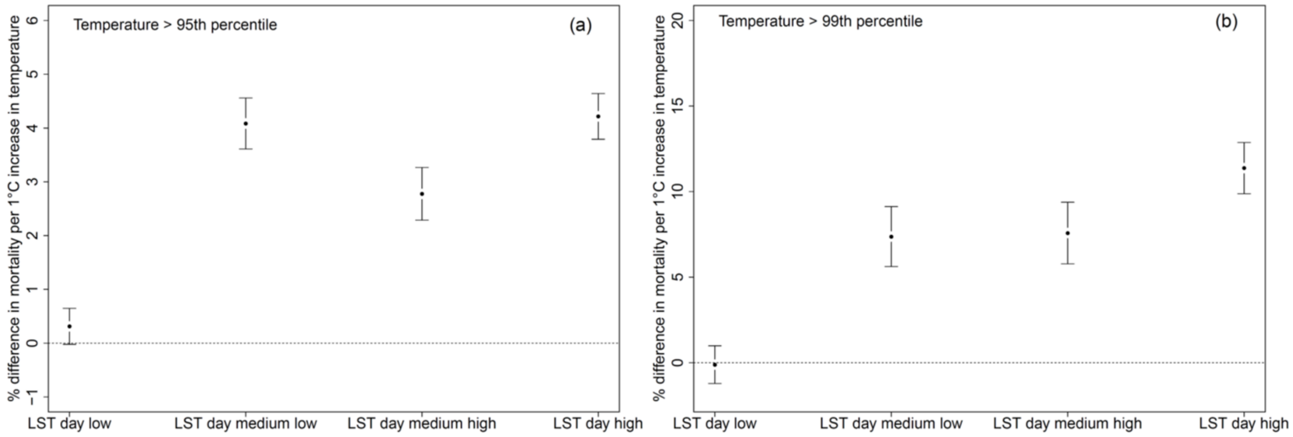

Figure S6: Difference in mortality increase among those > 65 years of age with a 1°C increase in UTCI above the 95th (a) and 99th (b) percentiles lags 0-2 for daytime LST quartiles with 95% confidence intervals. GAMs allowing for interaction between UTCI and different daytime LST classes were adjusted for long-term and seasonal trend (6 df per year), daily averages of O_3 and PM_{10} (lag 0-1), percent of parish population >65 years of age, building density, proportion of college graduates and percentage of population receiving social benefits. Daytime LST quartiles were 32.3°C, 33.8°C and 35.0°C.
